# UK best practice recommendations for children and young people <18 years with pre‐stage 3 type 1 diabetes, on behalf of the British Society for Paediatric Endocrinology and Diabetes (BSPED)

**DOI:** 10.1111/dme.70117

**Published:** 2025-09-23

**Authors:** Rachel E. J. Besser, Fiona Campbell, Katharine Damazer, Daniela Elleri, Kathleen M. Gillespie, Clare Hambling, Rebecca Martin, Fulya Mehta, Sarinda Millar, Pooja Sachdev, Tracy Savory, Ambika Shetty, Rabbi Swaby, Tabitha Randell, the BSPED

**Affiliations:** ^1^ Centre for Human Genetics, Nuffield Department of Medicine, NIHR Oxford Biomedical Research Centre, University of Oxford Oxford UK; ^2^ Children's Diabetes Centre Leeds Children's Hospital Leeds UK; ^3^ Oxford University Hospital Children's Psychological Medicine Oxford UK; ^4^ Royal Hospital for Children and Young People Edinburgh UK; ^5^ Diabetes and Metabolism Bristol Medical School University of Bristol Bristol UK; ^6^ Bridge Street Surgery Norfolk UK; ^7^ University College London Hospitals NHS Foundation Trust London UK; ^8^ Department of Paediatric Endocrinology & Diabetes Alder Hey Children's NHS Foundation Trust Liverpool UK; ^9^ Southern Health and Social Care Trust Portadown Northern Ireland UK; ^10^ School of Medicine University of Nottingham Nottingham UK; ^11^ Nottingham Children's Hospital Nottingham University Hospitals NHS Trust Nottingham UK; ^12^ PPI Representative and PPI Chair UK Islet Autoantibody Registry Oxford UK; ^13^ Noah's Ark Children's Hospital for Wales Cardiff and Vale University Health Board Cardiff UK

**Keywords:** diabetic ketoacidosis, monitoring, preclinical, screening, type 1 diabetes

## Abstract

Screening for childhood type 1 diabetes (T1D) is increasing worldwide. Historically, screening has been undertaken through research programmes, but increasingly in the UK, children and young people are also being tested in clinical care. This identifies children before the onset of clinical disease through measurement of four islet autoantibodies (IAb): anti‐glutamic acid decarboxylase; anti‐insulin; anti‐IA2 tyrosine phosphatase; and anti‐zinc transporter‐8. Otherwise well individuals confirmed to have ≥2 IAb have early‐stage T1D, meaning that they are in the pre‐symptomatic phase of the disease. This is categorised into stages, where stage 1 indicates ≥2 IAb and normoglycaemia, and stage 2 the presence of ≥2 IAb and dysglycaemia. Stage 3 T1D indicates that the diagnostic threshold for T1D has been reached, which may occur with or without symptoms of diabetes.

The goal of screening and monitoring programmes is to reduce the adverse clinical consequences of diabetic ketoacidosis at diagnosis and to identify children who may benefit from disease‐modifying therapies to delay or reverse progression to insulin requirement. Additional benefits include avoiding hospitalisation and preparation for the 'softer landing' into T1D. To seek these benefits, children should be monitored; yet many individuals decline follow‐up in a research context. We therefore describe a pathway suitable for children identified from both screening programmes and clinical care settings.

The pathway consists of 5 themes (IAb confirmation, monitoring of individuals in early‐stage T1D, starting insulin, monitoring in single IAb positivity, and audit standards against which the pathway can be assessed during implementation).

**What's new?**

- A UK care pathway covering diagnosis, monitoring, and starting insulin in early‐stage T1D.
- Support materials for Parents.
- Audit standards against which the pathway can be assessed during implementation.

## 1 BACKGROUND

### 1.1 Type 1 diabetes stages

**FIGURE 1:**
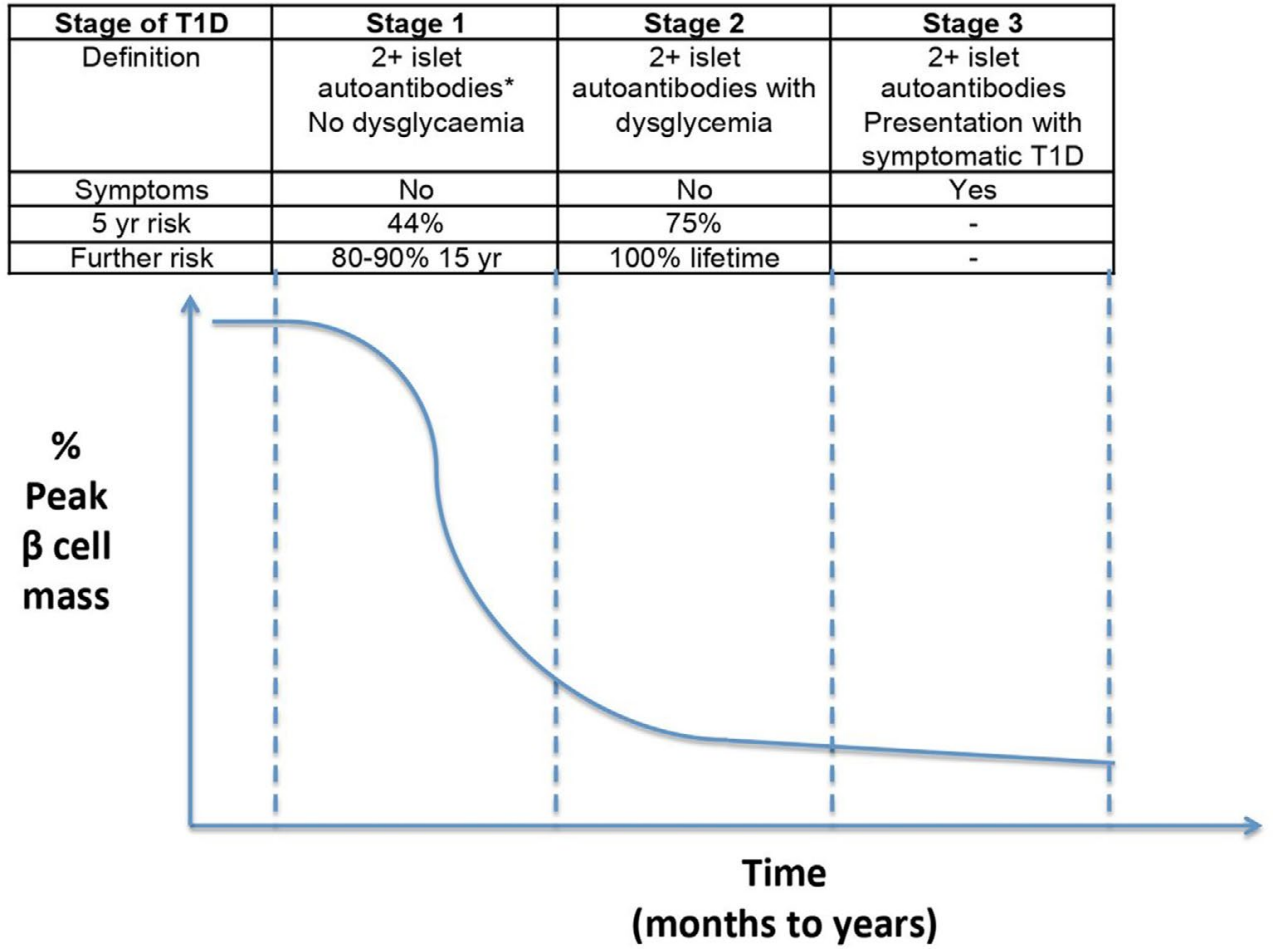
Stages of diabetes, taken with permission from,^2^ adapted from Insel et al.^1^ *Islet autoantibodies include anti‐insulin, glutamate decarboxylase, islet antigen 2 and islet‐specific zinc transporter. T1D, type 1 diabetes.

T1D can be identified before symptoms arise by detecting the presence of islet autoantibodies (IAb) to insulin (IAA), glutamic acid decarboxylase (GADA), IA2 tyrosine phosphatase (IA‐2A) and zinc transporter‐8 (ZnT8A). The presence of ≥2 IAb indicates that the T1D autoimmune process has begun, with classification into 3 distinct metabolic stages (Table 1)^1^; Figure 1:

- Stage 1 (normoglycaemia)
- Stage 2 (dysglycaemia)
- Stage 3 (hyperglycaemia)

**TABLE 1:** T1D Staging criteria, adapted from.^3^

Stage		Standard staging criteria			Other criteria
	Number of islet autoantibodies	FPG (mmol/L)	120‐min OGTT glucose (mmol/L)	HbA1c (mmol/mol)	
At risk	Single/transient single IAb positive	<5.6	<7.8	<39	–
1	≥2	<5.6	<7.8	<39	–
2^a^	≥2	5.6–6.9	7.8–11.0	39–47 OR ≥10% rise from the first measurement with stage 2	OGTT glucose at 30, 60 or 90‐min ≥11.1 mmol/L CGM^c^ >7.8 mmol/L for >10% time over 10 days of continuous CGM wear and confirmed by ≥1 non‐CGM glucose test listed
3^b^	≥1	≥7.0	≥11.1	≥48	A single random venous glucose ≥11.1 mmol/L with symptoms Two random venous glucose ≥11.1 mmol/L without symptoms CGM^c^ >7.8 mmol/L for >20% time over 10 days of continuous CGM wear and confirmed by ≥1 non‐CGM glucose test listed

^a^ Stage 2 T1D: at least 2 criteria or meeting the same criteria on two occasions.

^b^ Stage 3 T1D: Persistent hyperglycaemia with or without symptoms, as measured and confirmed by one or more of the listed criteria. In the absence of symptoms, stage 3 T1D requires confirmatory testing.

^c^ CGM is not included in the current ADA or ISPAD guidelines for staging criteria in T1D. If used, ideally CGM should be blinded and interpreted by a trained HCP.

### 1.2 Identifying individuals at risk of progression to clinical T1D

Genetically at‐risk children with ≥2 IAb (stage 1 or 2 T1D) have an 80%–90% risk of progression to clinical disease within 15 years.^4^, ^5^ The time to develop stage 3 T1D varies and is influenced by a number of factors.^5^, ^6^, ^7^, ^8^, ^9^ Progression rates are highest in children who develop IAb before the age of 10 years, particularly in those aged under 3 years and those with IA‐2A positivity (Table 2).^5^, ^9^, ^10^ Elevated body mass index may also be a risk factor for progression.^16^, ^17^

**TABLE 2:** Risk factors for progression to insulin requirement.

	Characteristics	Information gained
Age	≤10 years	The younger the age of first IAb detected, and the first 2 years after detection, the higher the risk of progression^5^, ^10^
Antibodies	≥2 islet autoantibodies^4^, ^5^ Positive IA‐2A^6^, ^7^, ^11^, ^12^	≥2: 84% 15 years risk of progression ≥3: 92% 15 years risk of progression
HbA1c	HbA1c ≥41 mmol/mol on 2 consecutive samples,^13^ or HbA1c ≥10% rise (even below 48 mmol/mol) on two consecutive measures 3–12 months apart^13^, ^14^	Median time to insulin 12 months since the observed rise^13^, ^14^
CGM	≥10% above 7.8 mmol/L	Median time to insulin 12 months^15^

Children with a single IAb are at lower risk (10%–15% risk of progression over the subsequent 15 years),^4^, ^5^ see 3.4, requiring a different follow‐up pathway. It is therefore important that if one IAb is detected (or test‐positive on islet cell antibody [ICA] or 3‐screen ELISA), that individual IAbs are measured, as this will influence the follow‐up pathway.

### 1.3 Potential benefits and harms of screening for type 1 diabetes

Screening programmes offering IAb testing and monitoring have demonstrated improved patient‐related outcomes, including reductions in the risk of diabetic ketoacidosis (DKA), a lower presenting HbA1c, with fewer individuals admitted to hospital at diagnosis.^9^, ^18^, ^19^ Children identified in early‐stage T1D (and their parents) can be offered support, gradual education and preparation to allow clinical T1D management to begin in a planned way, a so‐called 'softer landing' into insulin treatment. This is also an opportunity to offer preventative agents (if and when available) and recruitment into trials, which is an ongoing focus for research.^20^ Teplizumab (an anti‐CD3 therapy) has been approved in the United States by the Food and Drug Administration for the treatment of children 8 years and over, and adults, with stage 2 T1D. Teplizumab has been shown to delay T1D by median 2–3 years.^21^, ^22^ The Medicines and Healthcare products Regulatory Agency (MHRA) has licensed teplizumab for adults and children aged 8 years and over in stage 2 T1D. Access to teplizumab for use in the UK is currently under assessment by the National Institute of Clinical Excellence (NICE).

As may be expected, there is an increase in psychological distress (anxiety, depression) when parents (particularly maternal caregivers) are given the news that their child is IAb positive^9^, ^33^; although in the Fr1da study, psychological distress (depression) seen in parents of IAb positive children returned to baseline within 6–12 months.^9^ However, in the TEDDY study, anxiety levels in parents of children identified as genetically at‐risk only reduced if they were informed that their child was IAb negative.^33^ Dropout rates after the first IAb screen can be as high as 50%.^34^, ^35^, ^36^ The benefits of screening programmes (DKA reduction) are only seen in the context of active monitoring, rather than screening alone.^36^ A monitoring programme that is practical and encourages engagement is crucial. It is unclear what the long‐term effect is of living with the knowledge of risk, for example, the impact on lifestyle, behaviour and mood; and research is underway to address these knowledge gaps.^37^

## 2 INTRODUCTION TO THE PATHWAY

**FIGURE 2:**
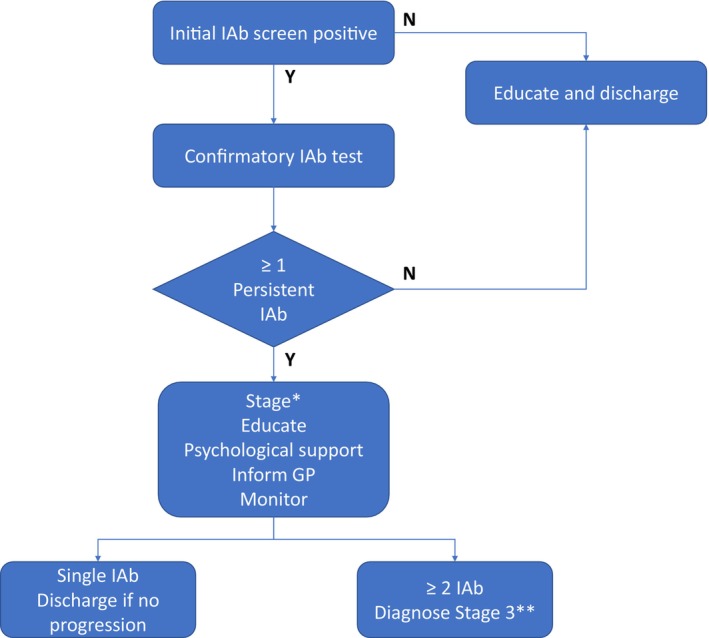
Overview of screening and monitoring pathway. *Metabolic staging only applies to individuals with 2 or more islet autoantibodies. The monitoring pathway is outlined in detail in Figure 3. **The diagnosis of stage 3 is according to criteria outlined in Table 1.

This pathway is for managing children and young people (CYP) identified with positive IAb but who have not yet been started on insulin therapy. We include a step‐by‐step clinical care pathway that covers diagnosis and IAb confirmation, the first clinic appointment, follow‐up and commencement of insulin therapy in CYP transitioning to clinical disease (Figure 2). We also include a pathway for individuals positive for a single IAb. A key objective is to educate healthcare professionals and provide simple, practical and actionable advice, with clear decision points, suitable for use in a United Kingdom (UK) healthcare setting. We, as a group of paediatric healthcare professionals across the devolved four nations of the UK, covering representation from medical, nursing, psychology, primary and secondary care, NHS England and parent representation, provide a pathway that accounts for the UK clinical context. We also provide audit standards against which the pathway can be assessed and may also be of use to commissioners and policymakers for future discussions on implementation and service provision.

The recommendations set out are adapted from the International Consensus Guidance for Monitoring Individuals with Islet Autoantibody‐Positive Pre‐Stage 3 Type 1 Diabetes (T1D).^3^ These follow on from other consensus statements.^23^, ^38^, ^39^, ^40^ We acknowledge that in the UK, most CYP with T1D are managed in secondary care, but primary care also plays a crucial role in the identification of CYP with new‐onset T1D. The pathway is suitable for CYP identified incidentally with IAb, as reported recently,^41^ or through a screening programme.

This pathway will be updated in light of emerging evidence to ensure the recommendations are evidence‐based and implementable. This document should therefore be regarded as a ‘live document’' and feedback is welcomed.

### 2.1 Definitions

At risk of T1D: single or transient single IAb.

Early‐stage T1D: defined by the presence of 2 or more IAb to IAA, GADA, IA‐2A, or ZnT8A, recorded ideally from two separate samples.

## 3 PATHWAY OVERVIEW: A PRACTICAL GUIDE

The pathway consists of 5 themes (IAb confirmation and diagnosis, monitoring of individuals in early‐stage T1D, starting insulin, monitoring in single IAb positivity, and audit standards against which the pathway can be assessed during implementation).

### 3.1 Theme 1: IAb confirmation and diagnosis

Once a child is identified as being IAb positive, through research or clinical care, this needs to be confirmed within 3 months, to (a) reduce false positives, and (b) determine the presence and persistence of single or multiple IAb status, which will inform the follow‐up pathway. Confirmatory testing should ideally be in a second sample (the ‘rule of twos’ outlined in^3^). This confirmatory testing should be in an established laboratory with cut‐offs suitable for the general population reference standards. In the UK, this is available through the UK Islet Autoantibody Registry, UKIAbRegistry.^37^ However, a child identified as having ≥2 IAb on initial testing should be offered follow‐up before confirmation if a significant delay is expected on confirmatory testing. Reversion is relatively uncommon in children with confirmed multiple IAb,^42^ and whilst progression to stage 3 T1D is slower in those who revert, progression is still present; therefore, follow‐up should continue.^43^

#### 3.1.1 Notes about other antibody measures

- ICA (multiple antigens, undefined; subjective measure), positives need to be confirmed in individual assays.
- 3 screen Elisa: a good first‐line screening test for GADA/IA‐2A/ZnT8A, but positives need to be confirmed in individual assays.

### 3.2 Theme 2: Monitoring of individuals in early‐stage T1D (the child or young person with 2 or more Islet autoantibodies)

#### 3.2.1 The First Clinic appointment

Priorities should include confirmatory testing (3.1), metabolic staging, choice of monitoring, and support.

#### 3.2.2 Metabolic staging

Staging is important to provide information to families on the expected rate of progression (Figure 1) and to inform frequency and options for follow‐up. In the research setting, the oral glucose tolerance test (OGTT) is used to stage T1D.^1^ When combined with other metrics (such as age, gender, and IA‐2A status) to create composite scores, it can improve information on the risk of progression.^1^, ^11^, ^24^, ^25^, ^26^, ^27^, ^28^, ^29^ In clinical practice, this may not be feasible or necessary if the goal is DKA prevention and preparation for insulin therapy. When this applies, a random venous glucose / HbA1c can be measured (Table 1). In the research setting, including assessing eligibility for a drug intervention to delay disease onset, accurate staging is needed.

**A capillary glucose in isolation should not be used to confirm clinical/stage 3 T1D: Table** 1.

#### 3.2.3 Practical steps

GP: Flag 'at risk' status on the primary care electronic health record; add SNOMED code (1290118005). Consider adding a “pop‐up” on the clinical record to highlight this risk when a child presents in primary care.

#### 3.2.4 Paediatric diabetes multidisciplinary team (MDT)

- Offer of support from diabetes/ paediatric psychologist to manage parental/ child anxiety
- Provide educational materials to parents and CYP (see Parent template letter, Appendix A)
- Flag at‐risk status on hospital electronic record to recommend that a child has a glucose measured if they attend hospital for any reason
- Educate about signs and symptoms of T1D
- Provide home capillary blood glucose and ketone meter; provide education on its use and safety net advice (see 3.2.6)
- Perform capillary glucose/HbA1c in clinic and explain its meaning
- Establish metabolic stage ‐ may require venous sample
- Communicate risk and plan follow‐up (see My Monitoring Plan, Appendix B)
- Communicate risk to primary care
- Refer to UK Islet autoantibody registry^37^

#### 3.2.5 Follow‐up: options for monitoring

##### Recommended follow‐up schedule by age and stage (Figure 3, Table 3)

**FIGURE 3:**
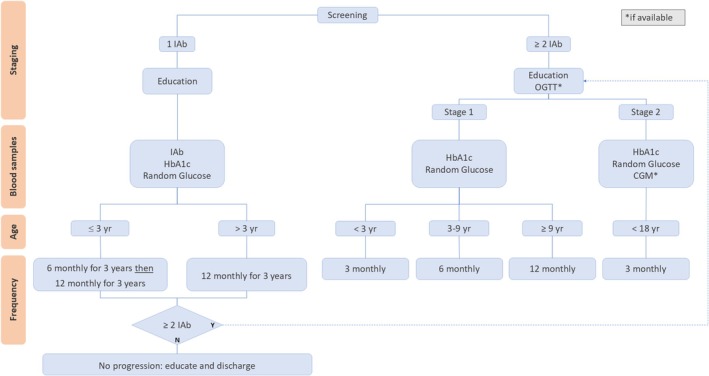
Follow‐up pathway for children with positive islet autoantibodies, adapted from Haller et al.^38^ Clinic appointments are recommended according to the frequency of follow‐up. IAb = islet autoantibody to IAA, GADA, IA‐2A and ZnT8A. OGTT = oral glucose tolerance test.

Clinic appointments are recommended according to the frequency of follow‐up (Figure 3). There are various modalities available for follow‐up depending on individual preference, locality, and resource availability (Table 3). The mainstay is likely to include random venous glucose, self‐monitoring blood glucose (SMBG) and HbA1c. Real‐time continuous glucose monitoring (CGM) used intermittently can be offered in stage 2 or early‐stage 3 T1D; however, unblinded use has not been validated for this purpose. There is also currently no universally agreed‐upon funding stream available for this in the UK NHS. The OGTT has been used in research monitoring programmes, but is not always well tolerated,^34^ and less invasive alternatives can be used if the purpose of testing is DKA prevention in clinical care.

**TABLE 3:** Options for staging and follow‐up in stage 1 and 2 T1D, adapted from.^23^

Metric	Advantages	Disadvantages	Use
OGTT	Gold standard	Frequent blood draws Poor reproducibility Requires prompt sample handling and analysis	To stage disease and predict progression; can be combined with other metrics to provide more accurate risk assessment scores^1^, ^11^, ^24^, ^25^, ^26^, ^27^, ^28^, ^29^
Random venous glucose	Single sample	Blood draw	Provides similar information to 2‐hour OGTT glucose^30^
HbA1c	High specificity	Poor sensitivity	Venous or capillary Information on risk of progression to insulin therapy: ≥41 mmol/mol or >10% rise on 2 consecutive measures 3–12 months apart^13^, ^14^, ^31^
CGM	Real‐time monitoring	Frequency and duration of wear uncertain; blinding not readily available and acceptability unknown	Information on risk of progression to insulin therapy; >10% >7.8 mmol/L^15^ Information on stage 3 T1D; >20% >7.8 mmol/L^32^
SMBG	Simple to use, provides immediate result	False positives Optimal frequency of testing uncertain	Immediate result

##### Random venous glucose

A random venous glucose can be collected at the same time as measurement of HbA1c. In one study, a cut‐off of ≥7.8 mmol/L was associated with a median time to stage 3 T1D of 1 year.^30^ If taken 2 h post‐meal, it can provide similar predictive characteristics to a 2‐h post‐OGTT venous glucose.

##### HbA1c

Multidisciplinary diabetes teams are familiar with the use of HbA1c, which provides a practical and easily accessible tool to measure glycaemic exposure. An HbA1c value is relatively insensitive and is influenced by red cell turnover. It may lag behind true hyperglycaemia, so it is not advised to use HbA1c in isolation for monitoring. However, repeated HbA1c assessment can indicate progression and can be a useful and minimally invasive method that also allows for home collection. In children who are progressing, HbA1c can rise within the non‐diabetes range up to 2 years before clinical onset. It has been demonstrated that an HbA1c ≥ 10% rise on 2 consecutive measures (3–12 months apart) or HbA1c ≥ 41 mmol/mol indicates a risk of progression to clinical disease by a median of 12 months.^13^, ^14^, ^31^ This metric is now included in the staging criteria (Table 1).

This can be a helpful window to start diabetes education, which can be individualised by members of the multidisciplinary team according to need.

##### SMBG

If a venous sample is not possible or practical, a capillary glucose sample is recommended. Home glucose monitoring is a practical, low‐cost method which can empower families in home monitoring, although the evidence base is limited.^44^ We recommend that all children with ≥2 IAb should be provided with a home glucose and a ketone meter, and provided with education in its use, frequency, and safety netting limits (see Appendix B, My monitoring plan).

###### Timing for SMBG

Ideally, SMBG should be taken 2 hours after a carbohydrate‐rich meal as post‐prandial glucose excursions are typically seen early. As some children can progress rapidly after developing IAb, SMBG should be measured more frequently after the first clinic visit to ensure progression is not imminent; e.g. once every 2 weeks for 4 weeks, then monthly for 3 months. If the glucose remains stable in stage 1 (2 h post‐meal under 8 mmol/L), SMBG can then be undertaken at times of illness, every 1–3 months, and according to the schedule in Figure 1.

As children with ≥2 IAb can have intermittent and significant hyperglycaemia at the time of illness, regular home glucose testing is also needed at times of illness or if there are signs/symptoms of diabetes mellitus.

###### Safety netting (hospital care)

####### Post‐prandial

BG >14 mmol/L, check ketones – if BG >14 mmol/L with symptoms or with ketones >0.6 mmol/L seek same‐day medical attention in hospital. If no ketones, call clinical team within working hours.

BG 11.1–14 mmol/L, start regular monitoring; if no ketones, call clinical team within working hours.

BG 8–11 mmol/L, start testing three times per week, inform clinical team non‐urgently – consider if this is stage 2; consider repeat HbA1c and random venous glucose.

####### Safety netting (primary care)

An SMBG recorded as ≥11.1 mmol/L in primary care needs a same‐day referral to a paediatric diabetes specialist in secondary care.

##### CGM

###### Indication

- Stage 2 T1D, to identify children progressing to insulin requirement. Greater than 10% time with glucose >7.8 mmol/L suggests 80% of individuals will progress to insulin requirement in 12 months.^13^, ^14^, ^15^ Local teams may wish to use this time to start diabetes education.
- Stage 3 T1D, to inform how to start insulin (insulin type and timing) (see 3.3)

Where CGM is felt to be clinically useful and where locally available on the NHS, CGM can be considered as an option in monitoring. However, whilst practical, the evidence base and acceptability of CGM use, in particular without blinding, and in general population children, needs further research. Cut‐offs are not universally agreed (Table 1) and have been derived from blinded use. The use of unblinded wear may influence behaviour, in particular food intake; therefore, the established cut‐offs may not apply. Using CGM has the potential to exacerbate anxiety. It is essential that education in its use is provided. Real‐time CGM used intermittently may have value for use in stage 2 to inform families/HCPs about relative time to progression, and in early stage 3 to inform type and timing of insulin treatment.

### 3.3 Theme 3: Starting insulin

When children are identified with early‐stage T1D, the majority will be in stage 1 (normoglycaemia). For those in stage 1 (normoglycaemia) or stage 2 (dysglycaemia), monitoring will reveal evolving hyperglycaemia. Insulin is not required in stage 1 or stage 2.

#### 3.3.1 Confirmation of stage 3 T1D

To confirm stage 3 T1D, individuals should meet recognised diagnostic criteria (see Table 1). Where symptoms are present, one diagnostic test for diabetes is needed; in the absence of symptoms, two are required.

A diagnosis of Stage 3 T1D should not be made solely on capillary glucose or CGM measures.

#### 3.3.2 Deciding when to start insulin treatment

In early‐stage 3 T1D, children may not have symptoms of hyperglycaemia. It may be difficult to assess osmotic symptoms of polyuria and polydipsia in very young children. Additional clinical features e.g. weight loss or declining exercise capacity should also be considered. The decision about when and how to start insulin therapy in the asymptomatic child is lacking in evidence and requires a holistic assessment accounting for the burden of insulin therapy, safety, and family choice.^45^ MDT involvement is needed to provide education and support.

We recommend an approach aimed at treating to target, according to suggestions from Besser and Griffin.^45^ Although not evidence‐based, this is extrapolated from evidence from the Diabetes Control and Complications Trial/Epidemiology of Diabetes Interventions and Complications (DCCT/EDIC) studies, which demonstrated that early glucose control has long‐term beneficial effects on renal and cardiovascular outcomes, the so‐called ‘'metabolic memory’' effect.^46^

**Confirmed stage 3 T1D**

Frequent and regular SMBG or continuous CGM should be started in an otherwise well and asymptomatic child.

It is important to gather data and not intervene with a one‐off test.

#### 3.3.3 Type of insulin

The type and timing of insulin treatment in early‐stage 3 T1D is lacking in evidence. The choice of initial insulin therapy should be tailored, escalated as clinically appropriate.^45^

Physiologically, children in early‐stage 3 T1D may be expected to lose their first‐phase insulin response initially, with a resultant glucose rise post‐prandially, often most pronounced after the largest meal intake or after the last meal of the day. This can be treated with prandial insulin.

Precise carbohydrate counting may not be achievable; children in early‐stage 3 T1D are often markedly insulin sensitive, needing only a small amount of insulin, above a carbohydrate threshold.

#### 3.3.4 Interaction with Primary care

A child presenting to primary care with a capillary glucose >11.1 mmol/L should be referred to paediatric diabetes services on the same day.

#### 3.3.5 Suggested roles of the diabetes MDT in supporting children and families with Stage 1–3 (“early‐stage”) T1D

Educational messages (educational materials required, under development)

- Explain the stages of T1D
- Discuss risk and pathway to diagnosis from early‐stage T1D to starting insulin
- Teach about home glucose and ketone monitoring/CGM, and normative values
- Explain signs and symptoms of T1D
- Provide parents with on‐call phone number and when to call
- Carbohydrate awareness
- Lifestyle /weight management, especially in those with elevated BMI SDS
- Other topics to discuss include: coping, guilt, adjustment, and preparation for life with T1D.

### 3.4 Theme 4: A child or young person with a single islet autoantibody

The rule of two's also applies to those individuals identified with a single positive IAb.

#### 3.4.1 Explanation for differences: single vs. multiple IAb status

The follow‐up pathway (Figure 1) for children confirmed to have a single positive IAb reflects the knowledge that, (a) the risk of progression is lower compared to those with ≥2 IAb (10%–15% vs. 84%)^4^, ^5^; (b) 50% revert to negative^42^, ^47^; (c) the highest risk of progression (10 of the 15%) occurs in the first 2 years after becoming IAb positive^5^; and in the youngest age group (under 5 years).^48^, ^49^ Progression is also higher for young children who are positive for single IA‐2A (40.5%), compared with GADA (12.9%) or IAA (13.1%).^5^

For young children, metabolic and autoantibody monitoring frequency in the first 2–3 years after first detection of an autoantibody is key, as this is when spread from at‐risk single to early‐stage T1D multiple IAb positivity is most likely, but also reversion to negative IAb.

After 3 years of monitoring, the IAb status predicts future risk.^4^ In the TEDDY study, among children with increased genetic risk for T1D (HLA class II genes), those who remained positive for a single IAb have a risk for T1D of 1.8 per 100 person years; children who revert to negative status have a risk of 0.14 per 100 person years, and children who have never been IAb positive have a risk of 0.06 per 100 person years.^42^ The rate of progression to multiple positive IAb status also declines with age.^50^

#### 3.4.2 Recommendations for monitoring of children positive for a single IAb (Figure 1)

- Children aged ≤3 years, monitor IAb status, random venous or capillary blood glucose and HbA1c every 6 months for 3 years; then annually thereafter for 3 more years. If no progression to development of multiple IAb, stop autoantibody and metabolic monitoring, and counsel for risk of clinical disease.
- Children aged >3 years at first positive test, monitor IAb status, random venous or capillary blood glucose and HbA1c annually for 3 years. If no progression to development of multiple IAb, stop autoantibody and metabolic monitoring, and counsel for the risk of clinical disease.
- For children with single IAb who revert to seronegative during monitoring, or do not progress, education should be provided, emphasising potential symptoms and awareness of DKA, and follow‐up can be discontinued.

### 3.5 Theme 5: Implementation and Audit standards

This is the first UK guideline recommending a clinical pathway for individuals identified before disease onset. We include proposed audit standards for implementation and against which this pathway can be assessed.

Suggested audit standards are:

1. Confirmation of islet autoantibody status from an approved laboratory
2. Education on the implications of a positive IAb status
3. Education on and provision of blood glucose and ketone monitoring equipment
4. Discussion of inclusion in the UK Islet Autoantibody registry
5. Adherence to monitoring recommendations (as in Figure 3) for:
  - CYP with 2 or more positive IAb
  - Children aged under 3 years at first positive test for single IAb
  - Children aged over 3 years at first positive test for single IAb
  - Children who revert to seronegative after a positive test for single IAb during monitoring
6. Number of IAb‐positive CYP who progress to stage 3 T1D per year
7. Number of IAb‐positive CYP who start insulin per year, including insulin regimen
8. Number of IAb‐positive CYP who develop DKA per year
9. Number of families of CYP with positive IAb accessing psychology support per year
10. Hospital admission at diagnosis of clinical T1D (ward/ intensive care/ high dependency)

## FUNDING INFORMATION

REJB is funded by the NIHR Oxford Biomedical Research Centre for this work. RS is funded by the Novo Nordisk UK Research Foundation through a research fellowship.

## CONFLICT OF INTEREST STATEMENT

REJB reports acting as an independent adviser for Provent Bio and received a speaking honorarium from Sanofi, which was donated to an education research fund. RS has received speaker honoraria from Sanofi.

## APPENDIX A Parent template letter

### A.1 Information for Parents with a child with 1 or more Type 1 diabetes antibodies

You might be feeling shocked, worried, or upset following the news that your child has type 1 diabetes (T1D) antibodies – these are also called islet autoantibodies. This information sheet provides more information about what it means for your child, including next steps for follow‐up from a healthcare professional and things to look out for as a parent.

### A.2 What is type 1 diabetes?

Insulin is a hormone (one of the body's chemical messengers) that controls blood glucose levels. In T1D, the immune system attacks part of the body called the pancreas, which makes insulin. Treatment involves replacing the body's insulin using injections or an insulin pump. Diabetes cannot be cured, but we can teach children, young people, and their families to manage it so that diabetes will not stop you/them from leading a full and active life.

### A.3 What are type 1 diabetes antibodies?

These are markers found in the blood which indicate that a child is more likely to get T1D. They can be found in the blood months or years before symptoms of T1D appear.

### A.4 Type 1 diabetes antibodies ‐ what do they mean?

Low risk: If one T1D antibody is found, this does not mean your child will develop T1D in the future. There may be a slight increased risk compared to the general population.

High risk: If two or more T1D antibodies are found, this shows that your child is already in the early stage of T1D, although there may be no symptoms for months or years. Your child's body should still control its blood glucose level now, but insulin is likely to be needed in the future.

If your child has normal blood glucose levels (this is called normoglycaemia, or stage 1 T1D), there is an 80%–90% chance of your child needing insulin before the age of 18 years and 100% during their lifetime. If your child already has abnormal blood glucose levels (this is called dysglycaemia, or stage 2 T1D), the chance of them needing insulin is around 75% over the next 5 years.

### A.5 What can I do?

There is nothing you could have done differently to prevent your child from developing T1D antibodies. There is no need to modify your day‐to‐day behaviour. We recommend that your child follows a healthy lifestyle, which includes balanced nutrition, regular exercise, good sleep, and general physical and emotional well‐being.

You need to take action if your child develops symptoms of T1D. If parents and doctors act quickly if symptoms appear, your child can get a quick diagnosis and early treatment with insulin, avoiding your child becoming seriously ill at diagnosis.

The 4 most common symptoms of T1D are going to the toilet a lot, increased thirst, being more tired than usual, and losing weight. We would like you to be aware of these symptoms and to seek help immediately from your GP or your local hospital if your child shows any symptoms – please do not delay. If you have a home blood glucose meter, you should check your child's blood glucose level at home and seek medical help.

### A.6 Know the 4 Ts of T1D

However, sometimes children (especially if they are young) do not display classic symptoms (the “4 Ts”), so if your child is unwell for other reasons, please get their blood glucose levels checked on the same day. If you have a home blood glucose meter, you should check your child's blood glucose level at home and seek medical help.

Further information about the symptoms of T1D can be found on the enclosed flyer and on the Diabetes UK web page: https://www.diabetes.org.uk/Get_involved/Campaigning/Our‐campaigns/4‐Ts‐campaign/.

### A.7 What happens next?

Your GP should be contacted by your health team to let them know your child's T1D antibody result, so that if your child needs to see a healthcare professional for whatever reason, they should consider checking your child's blood glucose level.

We recommend that your child be followed up by a healthcare professional so that they can be monitored to see if they are progressing to T1D. How often will depend on their age, their number of T1D antibodies, and whether their blood glucose levels are in the normal range. Follow‐up may include a finger prick or blood test at your local hospital or wearing a glucose sensor.

Please discuss what follow‐up your child needs with your hospital team. You might be feeling shocked, worried, or upset following the news that your child has T1D antibodies. The team at the hospital is on hand to support you with this.

### A.8 Are there any treatments available to delay type 1 diabetes?

One treatment (an immune drug called teplizumab) has been approved to delay T1D onset in the United States for children aged 8 years and over with abnormal blood glucose levels. This drug is currently under assessment in the UK by NICE. To find out more, please follow this link: Teplizumab|DiabetesUK.

The best way to find out if a treatment is available or if there is a drug trial to prevent T1D is to register with the UK Islet autoantibody registry. You can find out more at (https://www.ukiab.org).

## APPENDIX B My monitoring plan

### B.1 My monitoring plan

**Table:**

**My diabetes stage**	
**Completed by**	
**Date**	
**My next follow‐up**	
**Emergency helpline**	
**Non‐emergency helpline**	

### B.2 Home blood glucose monitoring plan

**Frequency:** ………. **When to test:** …………………..

AND at times of illness AND if symptoms of type 1 diabetes (tiredness, weight loss, increased thirst, increased urination).

**Table:**

Blood glucose level (mmol/L)	Action to take
	
	
	
	

### B.3 Other tests due

**Table:**

Test name	Follow‐up (months)
HbA1c	
CGM	
OGTT	
